# Chronic Mild Hyperglycemia in GCK-MODY Patients Does Not Increase Carotid Intima-Media Thickness

**DOI:** 10.1155/2013/718254

**Published:** 2013-09-11

**Authors:** Stepanka Pruhova, Petra Dusatkova, Pavel J. Kraml, Michal Kulich, Zdena Prochazkova, Jan Broz, Jaroslav Zikmund, Ondrej Cinek, Michal Andel, Oluf Pedersen, Torben Hansen, Jan Lebl

**Affiliations:** ^1^Department of Pediatrics, 2nd Faculty of Medicine, Charles University in Prague and University Hospital Motol, 150 06 Prague, Czech Republic; ^2^2nd Department of Internal Medicine, 3rd Faculty of Medicine, Charles University in Prague and University Hospital Kralovské Vinohrady, 100 81 Prague, Czech Republic; ^3^Department of Probability and Mathematical Statistics, Faculty of Mathematics and Physics, Charles University in Prague, 186 75 Prague, Czech Republic; ^4^Department of Internal Medicine, 2nd Faculty of Medicine, Charles University in Prague and University Hospital Motol, 150 06 Prague, Czech Republic; ^5^Department of Pediatrics, 3rd Faculty of Medicine, Charles University in Prague and University Hospital Kralovské Vinohrady, 100 81 Prague, Czech Republic; ^6^Hagedorn Research Institute, 2820 Gentofte, Denmark; ^7^Institute of Biomedical Sciences, Faculty of Health Science, University of Copenhagen, 2200 N Copenhagen, Denmark; ^8^The Novo Nordisk Foundation Center for Basic Metabolic Research, Faculty of Health Sciences, University of Copenhagen, Copenhagen, Denmark; ^9^Faculty of Health Sciences, University of Southern Denmark, 2820 Odense, Denmark

## Abstract

*Aim.* GCK-MODY is an autosomal dominant form of diabetes caused by heterozygous mutations in the glucokinase gene leading to a lifelong mild hyperglycemia. The risk of macrovascular complications is considered low, but studies are limited. We, therefore, investigated the carotid intima-media thickness (CIMT) as an indicator of macrovascular complications in a group of patients with GCK-MODY. *Methods.* Twenty-seven GCK mutation carriers and 24 controls recruited among their first-degree relatives were compared, all aging over 35 years. The CIMT was tested using a high-resolution B-mode carotid ultrasonography. Medical history, anthropometry, and biochemical blood workup were obtained. *Results.* The mean CIMT was 0.707 ± 0.215 mm (mean ± SD) in GCK mutation carriers and 0.690 ± 0.180 mm in control individuals. When adjusted for age, gender, and family status, the estimated mean difference in CIMT between the two groups increased to 0.049 mm (*P* = 0.19). No difference was detected for other characteristics, with the exception of fasting blood glucose (GCK-MODY 7.6 mmol/L ± 1.2 (136.4 mg/dL); controls 5.3 mmol/L ± 0.3 (95.4 mg/dL); *P* < 0.0001) and glycated hemoglobin HbA_1c_ (GCK-MODY 6.9% ± 1.0%, 52 mmol/mol ± 10; controls 5.7% ± 0.4%, 39 mmol/mol ± 3; *P* < 0.0001). The frequency of myocardial infarction and ischemic stroke did not differ between groups. *Conclusion.* Our data indicate that the persistent hyperglycemia in GCK-MODY is associated with a low risk of developing diabetic macrovascular complications.

## 1. Introduction

GCK-MODY (GCK diabetes, glucokinase diabetes, or MODY2) is a monogenic condition caused by heterozygous mutations in the gene encoding glucokinase (*GCK*) [1]. It is characterized by chronic, lifelong, and mild hyperglycemia present from birth, and less than 50% of patients fulfil the criteria for overt diabetes. GCK-MODY is not associated with insulin resistance or dyslipidemia [2]. 

The increased risk of atherosclerotic vascular disease—as compared with healthy individuals without diabetes—is known in subjects with impaired glucose tolerance, patients with type 2 diabetes, and patients with metabolic syndrome [3, 4]. Noninvasive imaging techniques, such as carotid intima-media thickness (CIMT) measurements, may help to stratify this risk of atherosclerosis, as well as the risk of myocardial ischemia [5]: the CIMT has been shown to independently predict coronary events in type 2 diabetes and cardiovascular diseases [4]. However, the situation in GCK-MODY is likely to be different from that in type 2 diabetes: patients with GCK-MODY have increased fasting blood glucose and relatively low 2-hour post-OGTT blood glucose without other components of metabolic syndrome or insulin resistance [6]. Such a blood glucose profile has been shown to be associated with lower rates of cardiovascular mortality among patients with type 2 diabetes [7]. Importantly, Niskanen et al. [8] demonstrated that components of insulin resistance syndrome, including hyperinsulinemia after an oral glucose load, serum lipid abnormalities, and elevated blood pressure, are major determinants of CIMT in patients with diabetes. 

The risk of macrovascular complications in GCK-MODY is considered low, but the data are scarce. We aimed to evaluate the carotid intima-media thickness (CIMT) as an indicator of this risk in GCK-MODY patients aging 35 years or older and their unaffected relatives, who share a similar environment and lifestyle.

## 2. Materials and Methods 

We studied 27 patients from 20 Czech families with genetically confirmed GCK-MODY (age 35–75 years; median 46 years) and 24 unaffected family members (siblings, parents, and partners) representing the control group (age 35–79 years; median 50 years). Each of the 20 participating families contributed 1 to 3 patients with GCK-MODY and 1 to 3 control individuals matched by age and gender. Control individuals with fasting blood glucose more than 5.6 mmol/L (100 mg/dL) and/or with a known history of diabetes were excluded from the study. The identification of families with GCK-MODY has been reported previously [9, 10]. Informed consent was obtained from all study participants. The study protocol was approved by the Ethics Committee of the 3rd Faculty of Medicine, Charles University in Prague, Czech Republic. 

All study participants were examined in a fasting state. The structured assessment included a questionnaire, anthropometric examination and blood sampling for biochemical analysis. The laboratory methods used have been described previously [11].

High-resolution B-mode carotid ultrasonography (using Phillips iU22 ultrasound) was performed to measure the CIMT of the distant wall for 1 cm lengths of the carotid bifurcation and the internal carotid and right and left common carotid arteries. The mean CIMT values of 10 sites were combined in an unweighted average to produce an overall CIMT. The upper normal limit of CIMT was set to 0.7 mm [12, 13]. The patient history of coronary heart disease and ischemic stroke was obtained from medical records. All participants were further investigated with echocardiography and ECG.

The clinical and demographic characteristics of GCK mutation carriers and control individuals were compared using Welch's two-sample *t*-tests (continuous variables) and Fisher's exact tests (categorical variables). Mixed linear regression models with CIMT, blood pressure, and serum creatinine as outcomes were used to estimate and test the adjusted effects of GCK mutation status. Age, gender, and mutation status were included as fixed effects, and families were included as random effects. All analyses were performed using the R statistical package [14]. *P* value ≤0.05 was considered statistically significant.

## 3. Results

No significant differences in baseline characteristics were found between patients and control individuals, with the exception of fasting blood glucose (GCK-MODY 7.6 mmol/L, SD ± 1.2 (136.4 mg/dL); controls 5.3 mmol/L, SD ± 0.3 (95.4 mg/dL); *P* < 0.0001) and glycated hemoglobin HbA_1c_ (GCK-MODY 6.9% (SD ± 1.0), 52 mmol/mol (SD ± 10); controls 5.7% (SD ± 0.4), 39 mmol/mol (SD ± 3); *P* < 0.0001) (Table 1). The prevalence of smokers and hypertensive patients was similar in both samples (*P* = 1).

**Figure 1:**
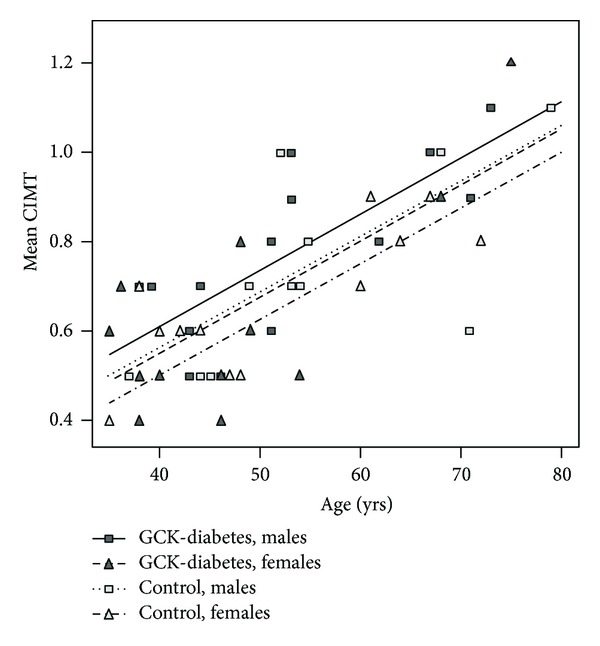
Estimated trends of mean CIMT by gender and GCK mutation status (adjusted for family).

The measured CIMT values for participants with and without GCK mutations are shown in Figure 1. The mean CIMT was 0.707 mm (range: 0.4–1.1) in GCK-MODY patients and was 0.692 mm (range: 0.4–1.1) in healthy control individuals. According to the published recommendations [12, 13], these values did not indicate an increased risk of accelerated atherosclerosis. After adjusting for age, gender, and family status, the estimated mean difference in CIMT between patients and healthy individuals increased slightly to 0.049 mm (95% CI from −0.026 to 0.123; *P* = 0.19). As expected, the estimated trends of mean CIMT indicated a moderate increase in CIMT with age and mutation status (see regression lines plotted in Figure 1). Carotid plaques (local intima-media thickening exceeding 1 mm and protruding into the lumen) were identified in 7 (25.9%) patients and 3 (12.5%) control individuals (*P* = 0.1), but all of these plaques were hemodynamically insignificant. 

Myocardial changes typical of ischemic heart disease described on echocardiography and/or ECG were detected in three of 27 patients with GCK-MODY and two of the 24 healthy control individuals (*P* = 0.866). Three study participants had suffered from myocardial infarction (two with GCK-MODY and one control individual) (*P* = 0.895) in the past, and two had suffered from ischemic stroke (one with GCK-MODY and one control individual). A similar proportion of participants (35%) from both groups were treated for hypertension with one or more antihypertensive drugs. Four of the 27 patients with GCK-MODY (14.8%) were treated with oral hypoglycemic agents, and one was treated with insulin. 

## 4. Discussion

To the best of our knowledge, the present study is the first case-control study including the CIMT measurements of GCK-MODY patients older than 35 years. The results are consistent with the mild natural course of GCK-MODY and confirm that GCK mutation is not associated with an increased risk of developing macroangiopathic complications. The 95% confidence interval of the CIMT difference, adjusted for age, family, and gender, is −0.026 to +0.123 mm, indicating that the possible increase in CIMT associated with GCK mutation is low and most likely clinically insignificant.

The absence of serious chronic microvascular complications in GCK-MODY was observed by Page et al. [15] and Velho et al. [16, 17], who described proliferative retinopathy in less than 4%, proteinuria in 6%, and peripheral neuropathy in 5% of patients with hyperglycemia at more than 5 years after diagnosis. A major problem with assessing diabetic complications in those patients is the differentiation between patients who only have GCK-MODY and those who develop type 2 diabetes in addition to GCK-MODY. It is generally assumed that patients with GCK-MODY do not necessarily develop insulin resistance or dyslipidemia in the natural disease course [2], and their glucose tolerance remains stable over many years [18]. Nevertheless, carrying a GCK mutation does not protect against the development of type 2 diabetes, which occurs at a similar prevalence in GCK-MODY patients and in the general population [19]. 

It has been reported that metabolic syndrome and insulin resistance are the major components of atherosclerosis risk in patients with diabetes and also in individuals without diabetes with insulin resistance [3, 20, 21], whereas the role of hyperglycemia in cardiovascular disease associated with type 2 diabetes is less clear [22]. In contrast with patients with type 2 diabetes, patients with GCK-MODY have mild hyperglycemia without other components of metabolic syndrome or insulin resistance [6]. Therefore, our study adds to the accumulating evidence that chronic mild hyperglycemia without additional components of metabolic syndrome has a milder effect on the development of macrovascular complications compared with the same glycemic levels associated with metabolic syndrome components. Admittedly, more subtle effects on CIMT would remain undetected, as the present study is moderately sized. The numbers of available patients in other studies are, however, comparable—a detailed analysis of the effects of p.Gly299Arg mutation in the GCK gene was limited to a single large pedigree [15], whereas another study reporting selected metabolic parameters and history data included 35 families, but these subjects were not examined at a single centre, and the data did not include CIMT [16, 17]. Thus, the number of participants may reflect a compromise between the depth of the acquired data and the subjects' willingness to undergo a complicated set of investigations. 

Patients with GCK-MODY exhibit only a small increase in glucose levels after oral glucose loading [2]. This might explain the observed lack of complications in GCK-MODY. By contrast, patients with type 2 diabetes have relatively high 2-hour glucose levels (as a proxy for postprandial glucose levels), indicating that postprandial glucose levels could be the most pathogenic glycemic factor for developing micro- and macrovascular complications. The CIMT has been shown to correlate more strongly with postprandial glycemia than with fasting hyperglycemia [23]. Additionally, the serum hs-CRP levels are lower in GCK-MODY patients than in patients with type 2 diabetes [24].

However, a common variant in the pancreatic GCK promoter has been shown to influence the risk of diabetes complications. März et al. showed [25] that the A allele at c.−30G>A of GCK was associated with an increased risk of coronary artery disease in not only patients with type 2 diabetes but also individuals who did not have diabetes, albeit with a much weaker association (OR = 1.27; 95% CI 1.02–1.59). Additionally, the SNP rs4607517, which is in linkage disequilibrium with c.−30G>A, has been associated with fasting glucose in genome-wide association studies. The association between components of the fasting glucose genetic risk score (represented by five SNPs, including rs4607517) and CIMT has been described with an increment of 0.0048 mm in carriers [26]. 

In conclusion, our data indicate that the natural course of mild lifelong hyperglycemia is associated with a low risk of developing diabetic macrovascular complications. However, patients with GCK-MODY should take steps to reduce the risk of developing “classical” type 2 diabetes in addition to GCK-MODY that is, avoid obesity and maintain a high level of physical activity. Our data support a conservative therapeutic approach for hyperglycemia in nonpregnant patients with GCK-MODY. Other risk factors for micro- and macrovascular complications should be treated according to present guidelines.

## Figures and Tables

**Table 1:** Clinical and biochemical characteristics of participants with GCK-MODY and control individuals.

	Controls	GCK-MODY	*P* value
	*n* = 24	*n = *27	
Gender			
Female	13 (54.2%)	12 (44.4%)	0.58
Male	11 (45.8%)	15 (55.6%)	
Smoking			
Nonsmoker	20 (83.3%)	22 (81.5%)	1
Smoker	4 (16.7%)	5 (18.5%)	
Age [yr]	53 (12.2)	49.8 (12.1)	0.35
Weight [kg]	81.7 (14)	77.2 (12.9)	0.23
Height [cm]	172 (7.43)	170 (7.34)	0.35
BMI [kg/m^2^]	27.7 (4.32)	26.8 (4.04)	0.43
Waist circumference [cm]	96.8 (13.6)	92.1 (12.3)	0.20
Hip circumference [cm]	108 (7.97)	104 (9.88)	0.081
Waist-to-hip ratio	0.892 (0.083)	0.881 (0.121)	0.73
Systolic blood pressure [mmHg]	124 (12.8)	122 (20.8)	0.8
Diastolic blood pressure [mmHg]	75.6 (9.7)	72.6 (12.5)	0.34
Glycemia [mmol/L], [mg/dl]	5.26 (0.33), 95.4	7.58 (1.17), 136.4	**<0.001**
HbA_1c_ [%]	5.74 (0.381)	6.92 (0.957)	**<0.001**
HbA_1c_ [mmol/mol]	39 (3)	52 (10)	**<0.001**
C-peptide [pmol/L]	871 (301)	853 (545)	0.89
Total cholesterol [mmol/L]	5.42 (0.866)	5.15 (0.856)	0.26
HDL cholesterol [mmol/L]	1.51 (0.368)	1.52 (0.366)	0.94
LDL cholesterol [mmol/L]	3.16 (0.66)	2.85 (0.757)	0.13
Triglycerides [mmol/L]	1.54 (0.858)	1.58 (1.33)	0.9
Creatinine [mmol/L]	70.5 (19.1)	73.6 (22.1)	0.59
GMT [*μ*kat/L]	0.468 (0.307)	0.647 (0.785)	0.28
Microalbuminuria [*μ*g/mg creatinine]	6.58 (10.1)	24.8 (61)	0.15
Albumin/creatinine ratio	0.818 (1.56)	5.11 (14.6)	0.17
Intima-media thickness [mm]	0.692 (0.189)	0.707 (0.215)	0.78

Numbers and percentages are shown for categorical variables; means and standard deviations are shown for numerical variables. *P* values compare the unadjusted means/percentages between the two groups. The bold font refers to statistically significant*P* values; *P* values less than 0.05 were considered as statistically significant.
